# In Vitro and In Vivo Evaluation of rPET/Cu-Alg Nanofibers for Anti-Infective Therapy

**DOI:** 10.3390/polym17010068

**Published:** 2024-12-30

**Authors:** Andreea Mihaela Grămadă (Pintilie), Adelina-Gabriela Niculescu, Alexandra Cătălina Bîrcă, Alina Maria Holban, Alina Ciceu, Cornel Balta, Hildegard Herman, Anca Hermenean, Alexandra-Elena Stoica, Simona Ardelean, Adina Alberts, Alexandru Mihai Grumezescu, Monica Puticiu

**Affiliations:** 1 Department of Science and Engineering of Oxide Materials and Nanomaterials, National University of Science and Technology Politehnica Bucharest, 011061 Bucharest, Romania; apintilie0503@stud.chimie.upb.ro (A.M.G.); adelina.niculescu@upb.ro (A.-G.N.); alexandra.birca@upb.ro (A.C.B.); oprea.elena19@gmail.com (A.-E.S.); agrumezescu@upb.ro (A.M.G.); 2 Research Institute of the University of Bucharest—ICUB, University of Bucharest, 050657 Bucharest, Romania; alina.m.holban@bio.unibuc.ro; 3 Faculty of Biology, University of Bucharest, 030018 Bucharest, Romania; 4 “Aurel Ardelean” Institute of Life Sciences, Vasile Goldis Western University of Arad, 310025 Arad, Romania; ciceu.alina@uvvg.ro (A.C.); balta.cornel@uvvg.ro (C.B.); herman.hildegard@uvvg.ro (H.H.); hermenean.anca@uvvg.ro (A.H.); 5 Faculty of Medicine, Vasile Goldis Western University of Arad, 310025 Arad, Romania; puticiu.monica@uvvg.ro; 6 Faculty of Pharmacy, Vasile Goldis Western University of Arad, 310025 Arad, Romania; ardelean.simona@uvvg.ro; 7 Carol Davila University of Medicine and Pharmacy, 050474 Bucharest, Romania

**Keywords:** polyethylene terephthalate nanofibers, electrospun membranes, recycled PET, copper alginate, antimicrobial activity, biocompatibility, wound dressing

## Abstract

With the growing interest in nanofibers and the urgent need to address environmental concerns associated with plastic waste, there is an increasing focus on using recycled materials to develop advanced healthcare solutions. This study explores the potential of recycled poly(ethylene terephthalate) (PET) nanofibers, functionalized with copper-enhanced alginate, for applications in wound dressings. Nanofibers with desirable antimicrobial properties were developed using chemical recycling and electrospinning techniques, offering a sustainable and effective option for managing wound infections and promoting healing. SEM and FT-IR analyses confirmed that the obtained nanofibers possess optimal physicochemical properties, including well-organized morphology, appropriate dimensions, and structural integrity. Biological evaluations revealed significant antimicrobial activity, with the materials effectively inhibiting microbial adherence and biofilm formation while maintaining good biocompatibility in both in vitro and in vivo studies. These findings highlight the potential of recycled PET-based nanofibers as advanced wound dressing materials to reduce infection risks and support tissue regeneration in clinical applications.

## 1. Introduction

Nanotechnology holds increasing promise as a discipline of science, being a frontier research topic for developing advanced materials with unique properties and enhancing economic competitiveness [1,2,3]. Among the plethora of possibilities regarding the morphology of nanotechnological products, nanofibers are comparatively new nanomaterials that exhibit exceptional attributes. When polymer fibers’ diameter is shrunk from micrometers to submicronic dimensions, the materials gain a high aspect ratio, superior mechanical performance, a high surface area/volume ratio, flexibility in surface functionalities, good permeability, extraordinary porosity with interconnectivity between pores in mats, and high potential to create composites with other materials [4,5,6,7,8,9]. Thus, utilizing nanofibers can greatly enhance various bulk materials’ electrical, optical, thermal, and mechanical features [10]. With their distinctive physicochemical properties, nanofibers have attracted interest for a wide range of applications, including medical textiles, tissue engineering scaffolds, wound dressings, drug delivery systems, and cosmetics [4,7].

Various materials, including metal, metal oxides, carbon, and polymers, can be chosen to fabricate nanofibers. Nevertheless, producing nanomaterials with such a unique one-dimensional structure, well-defined morphology, and high yield remains challenging [10,11]. Moreover, environmental concerns and the depletion of nonrenewable resources are driving up demand for sustainable and eco-friendly options as raw materials for nanofiber production [12]. Thus, there is a recent tendency to use recycled and biodegradable materials as a way to reduce environmental impact, promote green technology development, and ensure long-term economic benefits [13,14,15].

One interesting and feasible possibility for creating polymeric nanofibers is to use recycled polyethylene terephthalate (PET). This plastic material is widely available, being so commonly used for a broad range of applications that it has become one of the most frequent pollutants [16]. Inappropriate disposal of PET-based bottles, containers, and textiles has led to the increasing accumulation of plastic waste, which, due to its non-biodegradability and persistence, contributes to landfill overflow and marine pollution [17,18,19,20,21,22]. Therefore, recycling PET is essential to mitigate environmental issues and generate economic advantages. In addition to ecological considerations, using recycled PET also reduces the demand for virgin materials and diminishes production costs [22,23,24,25].

The topic of recycling PET for use in healthcare applications is of great global interest, especially in the context of finding economical solutions for the circular economy [26,27,28]. Nonetheless, several issues must be addressed before recycled PET can be widely adopted in such sensitive applications. The primary concerns are related to the potential presence of contaminants in the recycled material that may be transferred to the final recycled product and pose risks for medical use [29]. Moreover, the determination of long-term biocompatibility and stability of recycled materials in physiological environments must be considered to ensure the safety of these alternatives for various biomedical purposes [30].

To overcome these challenges and endow recycled PET-based fibers with superior properties, composite nanofibers can be used instead of pristine materials. Composite nanofibers (i.e., nanodimensional fibers that combine two or more materials) benefit from several advantageous features, including increased mechanical strength, improved thermal stability, multifunctionality, and synergistic activity between component materials [31,32,33]. To suitably functionalize recycled PET fibers for biomedical applications, this study proposes the addition of copper-enhanced alginate, aiming to synergistically combine the advantageous properties of the two materials and create distinct nanocomposites.

On the one hand, alginate, a natural polymer derived from brown seaweed, has been recognized for its biocompatibility, biodegradability, antibacterial potential, and ability to form hydrogels. These characteristics have made alginate attractive for various biomedical and industrial applications, including wound dressings, drug delivery systems, and tissue engineering scaffolds [34,35,36,37]. On the other hand, copper ions are known to exhibit antimicrobial properties attributed to a series of mechanisms, such as the generation of reactive oxygen species (ROS), disruption of bacterial cell membranes, interference with microbial enzyme systems, and damage to microbial DNA [38,39,40]. Numerous studies have shown that copper-infused materials limit microbial growth. Specifically, copper-coated surfaces and fabrics have been proven to drastically reduce rates of microbiological contamination and infection, supporting the use of copper as an antibacterial agent in various applications [38,41,42,43,44,45]. Thus, combining these three materials (i.e., recycled PET, alginate, and copper) can potentially create adequate and high-performing nanofibers for advanced healthcare solutions.

Differently categorized procedures can be used to approach nanofiber fabrication, such as top-down and bottom-up methods; physical, chemical, and biological techniques; and spinning and non-spinning methods [4,33,46]. Among existing possibilities, electrospinning is a versatile method employing the application of a high voltage to a polymer solution, which subsequently creates a charged jet that solidifies into fibers upon reaching a collector. Parameters such as the viscosity and conductivity of the polymer solution, the flow rate of the solution, the applied voltage, the distance between the needle and the collector, the humidity, and the temperature can be varied toward the aim of optimizing the quality and morphology of the nanofibers [33,46,47,48]. Thus, the simplicity, cost-effectiveness, scalability, and precise control over fiber diameter and morphology offered by the electrospinning method have increased its popularity in producing nanofibrous materials with enhanced performance and broad applicability [32,46,47,48,49,50].

In this context, this study utilized electrospinning to synthesize composite nanofibers composed of recycled PET and copper-enhanced alginate to create membranes with synergistic functionality. The obtained novel nanostructured materials were further characterized through a series of physicochemical and biological analyses, demonstrating their desirable morphostructural features, biocompatibility, and potent antimicrobial activity. By blending green technology with state-of-the-art biomaterials research, this work positions itself at the intersection of sustainable materials science and applied biomedical innovation.

## 2. Materials and Methods

### 2.1. Materials

All chemical reagents used in this study were of analytical grade and were obtained from Sigma Aldrich (Darmstadt, Germany). To ensure consistency and reliability in the experimental procedures, the reagents were used as received without any further purification.

### 2.2. Electrospinning Technique

The PET samples were prepared by dissolving 1 g of PET in a mixture of 20 mL dichloromethane and 2 mL trifluoroacetic acid. The solution was homogenized using a magnetic stirrer to ensure consistency. A 4.54% PET solution was then deposited onto aluminum foil using the electrospinning technique. The experiment was performed at four deposition rates: 10 mL/h, 7.5 mL/h, 5 mL/h, and 2.5 mL/h. The electrospinning process was carried out under the following parameters: an applied voltage of –5.73 kV at the nozzle and 17.53 kV at the collector, a heating power of 0.6 kW, a humidity level of 35%, and a temperature of 27 °C.

After deposition, the samples were carefully removed from the aluminum foil and cut into 1 cm × 1 cm sections. To functionalize the samples, two solutions were prepared: solution 1 consisted of 1% ALG (made by dissolving 1 g of ALG in 99 mL of distilled water), and solution 2 consisted of 1% CuCl_2_ (made by dissolving 1 g of CuCl_2_ in 99 mL of distilled water). The samples were first immersed in the ALG solution for 10 min under magnetic stirring, followed by immersion in the CuCl_2_ solution for another 10 min. Afterward, the samples were washed thoroughly with distilled water to remove any excess reactants and left to dry at room temperature.

The resulting samples were labeled as PET@ALG/Cu, with the label followed by a deposition rate (e.g., 10 mL/h, 7.5 mL/h, 5 mL/h, or 2.5 mL/h), representing the respective rates used during the electrospinning process.

### 2.3. Characterization

#### 2.3.1. Physicochemical Characterization

To investigate the morphology of the membranes based on recycled PET, the samples were analyzed using a scanning electron microscope (SEM) from FEI (Hillsboro, OR, USA). The images were obtained by capturing the resulting secondary electron beam at an accelerating voltage of 30 keV.

To investigate the integrity of the functional groups characteristic of the prepared membranes, a section of 1 cm × 1 cm was analyzed using a ZnSe crystal in a Nicolet 6700 FT-IR spectrometer from Thermo Nicolet (Madison, WI, USA). The measurements were performed at room temperature with 32 scans conducted in the range of 4000 to 600 cm^−1^ at a resolution of 4 cm^−1^. Data acquisition and processing were facilitated by connecting the spectrometer to a processing unit equipped with Omnic software (version 8.2, Thermo Nicolet).

Grazing incidence X-ray diffraction (GIXRD) was carried out using a Panalytical Empyrean diffractometer (PANalytical, Almelo, The Netherlands) equipped with CuKα radiation (wavelength: 1.541874 Å). The instrument included a 2× Ge (220) hybrid monochromator for Cu and a parallel plate collimator integrated with the PIXcel3D detector. The diffraction data were collected by scanning along the 2θ axis over a range of 5° to 80°, with an incidence angle set at 0.5°, a step size of 0.04°, and a time of 3 s per step.

#### 2.3.2. Evaluation of the Antimicrobial Effect

The microbial strains employed in this study were obtained from the Microbiology Laboratory, Faculty of Biology, University of Bucharest. The strains included *Staphylococcus aureus* ATCC 25923 (Gram-positive), *Pseudomonas aeruginosa* ATCC 27853 (Gram-negative), *Candida albicans* ATCC 10231 (yeast), and *Aspergillus niger* ATCC 16888 (filamentous microfungus). These strains were selected to evaluate the antimicrobial properties of the materials against a diverse spectrum of microbial species.

The effect of the prepared rPET samples on the growth of microorganisms in liquid media (planktonic cultures) was assessed by preparing samples of 1 cm × 1 cm sterilized with UV radiation for 30 min on each side. Each fragment was placed in a well of a sterile 6-well plate, to which 2 mL of liquid medium (simple broth for bacteria or liquid YPG for yeasts) and 20 μL of microbial suspension with a density of 0.5 McFarland (bacteria) or 1 McFarland (yeasts), prepared in sterile physiological saline, were added. The plates were incubated at 37 °C for 24 h. After incubation, 200 μL of the microbial suspensions were transferred to sterile 96-well plates, and the turbidity (absorbance) of the microbial cultures was measured spectrophotometrically at 600 nm.

To assess the effect of rPET samples on microbial adherence and biofilm formation, the samples were cut into 1 cm × 1 cm pieces and sterilized with UV radiation for 30 min on each side. The sterile fragments were placed individually in wells of a sterile 6-well plate containing 2 mL of simple broth and 20 μL of microbial suspension with a density of 0.5 McFarland (bacteria) or 1 McFarland (yeasts), prepared in PBS. The plates were incubated at 37 °C for 24 h, after which the materials were washed with sterile physiological saline, and the medium was replaced to allow biofilm development. The plates were incubated for 24, 48, and 72 h to evaluate adherence and biofilm formation. After each incubation period, the samples were washed with PBS and placed in sterile tubes containing 1 mL of sterile physiological saline. The tubes were vortexed for 30 s and sonicated for 10 s to detach cells from the biofilm. The resulting cell suspension was diluted, and different dilutions were inoculated onto solidified culture medium to quantify the number of colony-forming units (CFUs).

To assess antifungal activity, potato dextrose agar (PDA) medium was distributed in Petri dishes with a diameter of 10 cm. The surface of the medium was inoculated with a standardized spore suspension of *Aspergillus niger* prepared in PBS supplemented with 1% Tween 80. A 1 cm × 1 cm sterilized sample was placed in the center of each dish. The plates were incubated at room temperature for 4 weeks, with weekly inspections to monitor the appearance and integrity of the tested samples in the presence of fungal cultures.

### 2.4. In Vitro Experimental Design

#### 2.4.1. Cell Proliferation—MTT Method

The MTT assay (Vybrant MTT Cell Proliferation Assay Kit, Molecular Probe) was used as a quantitative colorimetric method to assess cell proliferation, viability, and cytotoxicity. This method is based on the reduction of the yellow tetrazolium salt MTT (3-(4,5-dimethylthiazol-2-yl)-2,5-diphenyltetrazolium bromide) to dark blue formazan by mitochondrial enzymes, primarily succinate dehydrogenase, reflecting cellular and mitochondrial integrity. The water-insoluble formazan crystals were solubilized using an SDS-HCl solution to allow spectrophotometric evaluation of optical density (OD), which correlates with the number of metabolically active cells in the culture.

Mesenchymal stem cells (AFSC cells) isolated from amniotic fluid were cultured in 96-well plates at a seeding density of 3000 cells per well under various experimental conditions in the presence of the obtained samples. After a 24 h treatment, 10 µL of 12 mM MTT solution was added to each well, and the plates were incubated at 37 °C for 4 h. Subsequently, 100 µL of SDS-HCl solution was added to dissolve the formazan crystals, followed by pipetting to homogenize the solution. The plates were incubated for 1 h to complete solubilization, and any bubbles that could interfere with optical readings were carefully removed. The absorbance of the samples was measured at 570 nm using a TECAN spectrophotometer (Männedorf, Switzerland).

#### 2.4.2. Evaluation of Cellular Oxidative Stress—GSH-Glo™ Glutathione Assay

To evaluate oxidative stress, AFSC cells were seeded in 96-well plates at a density of 3000 cells per well in 300 µL of DMEM supplemented with 10% fetal bovine serum and 1% antibiotics (penicillin and streptomycin/neomycin). Twenty-four hours after seeding, the cells were exposed to the biomaterials.

The GSH-Glo™ Glutathione Assay (Promega) was used to measure the levels of glutathione, a critical cellular antioxidant. Glutathione produced by the cells is enzymatically transformed into oxidized glutathione by glutathione S-transferase, and the extent of transformation is directly proportional to the enzyme’s activity. The assay involved adding 100 µL of 1X GSH-Glo™ Reagent to the wells, followed by incubation at 37 °C for 30 min. Subsequently, 100 µL of Luciferin Detection Reagent was added, and the plates were incubated for an additional 15 min at 37 °C. After incubation, the plates were homogenized, and luminescence was measured using a luminometer, providing insights into oxidative stress levels in the cells.

### 2.5. In Vivo Experimental Design

The in vivo experiment was performed on CD1 mice from the animal facility of the Vasile Goldis Western University of Arad, and the protocol was previously approved by the University’s Research Ethics Commission.

CD1 mice were housed in cages with controlled airflow and a standard 12 h light/dark cycle. Pre-sterilized materials (UV for 30 min) were implanted in a subcutaneous pocket in the dorsal region under intraperitoneal anesthesia. Mice were randomly assigned to 5 experimental groups (n = 20), as follows: Control, NanoAlg 10 mL/h, NanoAlg 7.5 mL/h, NanoAlg 5 mL/h, and NanoAlg 2.5 mL/h. Within each group, half were euthanized after 24 h and the rest at 7 days post-surgery. After implantation, the mice were housed individually. A veterinarian performed the daily clinical examination, according to the following parameters: appearance of surgical incision, redness, infection, edema, abscess, hematoma, and scars. Biopsies were performed, respectively, at 24 h and then 7 days after surgery, under anesthesia. Blood was also collected by cardiac puncture for biochemical analysis.

#### 2.5.1. Biochemistry

The collected blood was centrifuged at 3500 rpm for 10 min. Samples were analyzed for C-reactive protein (CRP) level evaluation on a Mindray BS-120 (ShenzenMindray Bio-Medical Electronics Co., Ltd., Shenzhen, China) chemistry analyzer using a CRP FL reagent kit (ChemaDiagnostica, Monsano, Italy).

#### 2.5.2. Histology

The biopsies (material together with surrounding tissues) were fixed in 4% paraformaldehyde solution, embedded in paraffin, sectioned at 5 μm, and stained with hematoxylin and eosin (H&E) and Masson–Goldner trichrome. The microscopic sections were analyzed under a microscope (Olympus BX43 equipped with an Olympus XC30 digital camera (Hamburg, Germany) and CellSens software, version 3.2). Sections were scored on the degree of inflammatory infiltrate, fibroblasts, and neovascularization. Each histometric parameter was graded on a scale of 0–4 for the amount of tissue reaction: - (not present) to ++++ (extensive).

#### 2.5.3. Immunohistochemistry

Immunohistochemistry was performed on 5 µm sections, deparaffinized and rehydrated using a standard protocol. Immunostaining was performed using a Novocastra Peroxidase/DAB kit (Leica Biosystems, Nussloch, mmunostaining was performed using a Novocastra PeGermany), according to the manufacturer’s instructions. The primary antibody used was polyclonal anti-TNF-α antibody at a dilution of 1:100 (Santa Cruz, CA, USA).

A negative control was generated by replacing primary antibodies with irrelevant immunoglobulins of matched isotype used under the same conditions as the primary antibodies. The slides were analyzed under an optical microscope.

#### 2.5.4. Immunofluorescence

After antigen unmasking with sodium citrate buffer (pH 6.0) and protein blocking in 1% bovine serum albumin (BSA) and 5% normal goat serum in PBS for 1 h, sections were exposed to primary antibody F40/80 (Abcam, Cambridge, UK, dilution 1:100). An Alexa Fluor dye-conjugated secondary antibody (1:500) was used, and nuclei were counterstained with DAPI. The fluorescence was visualized by confocal microscopy (Leica TCS SP8 confocal microscope).

## 3. Results

### 3.1. Physicochemical Characterization

**Figure 1:**
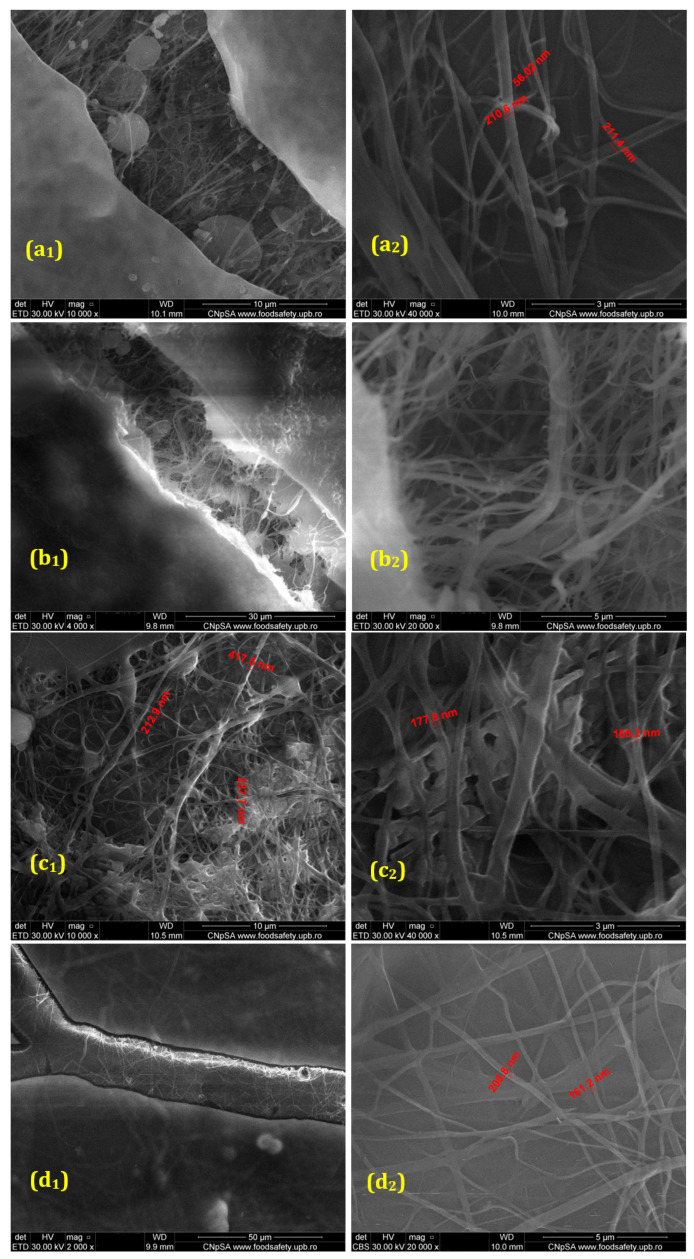
SEM micrographs of the PET@Alg/Cu samples, where (**a_1_**,**a_2_**)—10 mL/h; (**b_1_**,**b_2_**)—7.5 mL/h; (**c_1_**,**c_2_**)—5 mL/h; and (**d_1_**,**d_2_**)—2.5 mL/h.

The provided SEM micrographs (Figure 1) illustrate the main structural features of membranes produced through electrospinning, including recycled PET (control) and PET@Alg/Cu membranes.

The images confirm that the fibers are randomly oriented with significant curvatures along their axes, likely caused by the asynchronous deposition of fiber segments due to inherent instabilities such as asymmetric oscillations. The random orientation is further influenced by the repulsive electrostatic forces generated by surface charges. However, the use of a rotating collector introduces a slight alignment of the fibers, as indicated in the images. The fiber diameters range between 50 and 200 nm, with variations depending on the mass ratios used in the electrospinning process, as seen in the detailed measurements provided in the images.

For the PET@Alg/Cu membranes, the SEM images reveal a fibrous morphology similar to that of the PET membranes, with an additional coating of alginate on the fiber surfaces. This alginate layer is particularly evident in the detailed images, where a surface defect was intentionally created to analyze the coating’s structure and thickness. These observations confirm the successful integration of the alginate layer into the fibrous membrane architecture.

**Figure 2:**
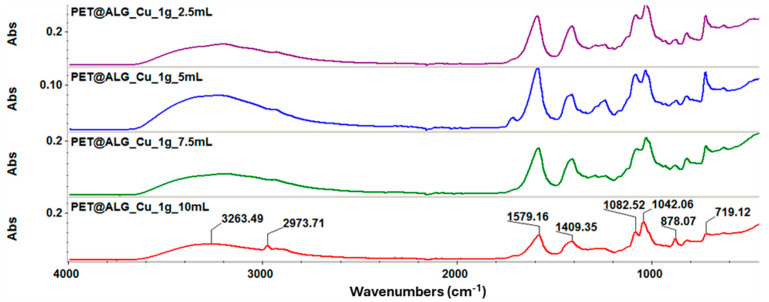
FT-IR spectra recorded for PET@Alg/Cu.

Figure 2 presents the results of the investigation conducted through infrared spectroscopy on PET@Alg/Cu membranes. The recorded spectra indicate that the prominent absorption bands are primarily attributable to the alginate layer covering the PET membranes. The characteristic bands of alginate include broad absorption in the range of 3263–3200 cm^−1^, associated with the OH group of the pyranose ring, and a band at 2973 cm^−1^ corresponding to the C-H bond. Additional bands can be observed at 1579 cm^−1^ and 1409 cm^−1^, corresponding to the carbonyl groups (C=O) and asymmetric stretching of the –O-C-O– group. The bands at 1082 cm^−1^ and 1042 cm^−1^ are attributable to C-O stretching vibrations, further confirming the presence of alginate on the PET surface. The absorption bands at 878 cm^−1^ and 719 cm^−1^ are characteristic of the pyranose structure of copper alginate.

Furthermore, the interaction between alginate’s carboxylate ions and copper ions is evidenced by the shifts in absorption bands, particularly in the 1409–1579 cm^−1^ range. These shifts highlight potential ionic interactions and hydrogen bonding between alginate and copper, which contribute to the material’s stability, promote a uniform alginate coating, and enhance its antimicrobial effectiveness.

**Figure 3:**
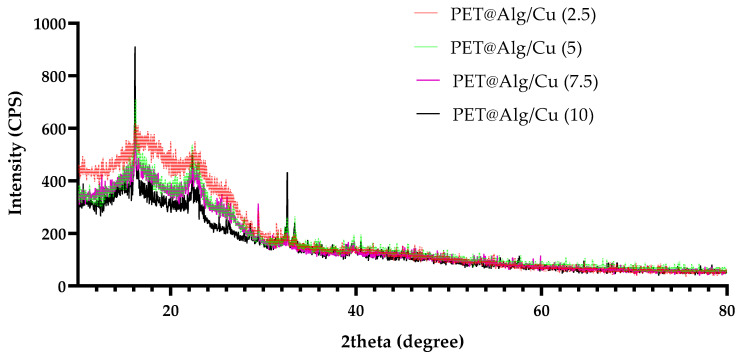
XRD patterns of electrospun PET coated with copper alginate at different flow rates (2.5, 5, 7.5, and 10 mL/h).

The XRD patterns of electrospun PET coated with copper alginate highlight the semi-crystalline nature of the PET substrate, with characteristic diffraction peaks observed in the patterns (Figure 3). A broad peak can be observed around 2θ = 16.0°−16.5°, which corresponds to the (0 −1 1) plane of PET, as reported in the literature. Additionally, a distinct double peak appears in the range of 2θ = 22°−23°, which is associated with the (−1 1 1) and (−1 1 0) planes, characteristic of the crystalline structure of PET. These assignments align with previous findings in studies of PET crystallinity, supporting the partially crystalline structure of the material [51,52,53].

### 3.2. Evaluation of Antimicrobial Effect

**Figure 4:**
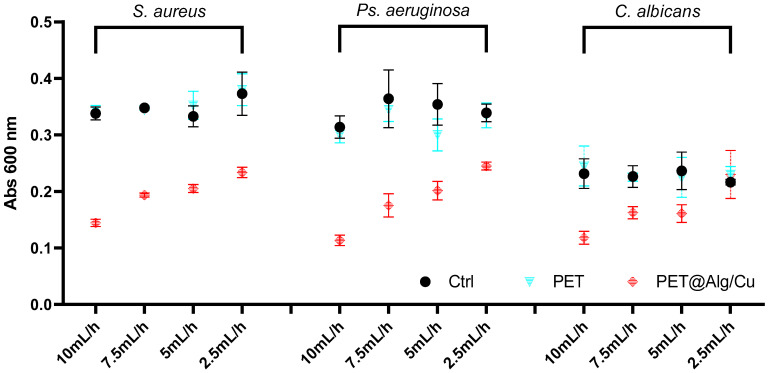
Graphical representation of absorbance values for planktonic cultures of *S. aureus*, *Ps. aeruginosa*, and *C. albicans* after 24 h in the presence of recycled PET-based materials (Ctrl, PET, and PET@Alg/Cu).

The antimicrobial effect of the tested materials was evaluated on planktonic microbial cultures, as shown in Figure 4. The results indicate that PET@Alg/Cu membranes consistently demonstrated the highest antimicrobial activity across all tested microbial species (*S. aureus*, *Ps. aeruginosa*, and *C. albicans*), outperforming the control and PET-only samples.

For *S. aureus*, *Ps. aeruginosa*, and *C. albicans*, the PET@Alg/Cu membranes exhibited significant reductions in absorbance values across all deposition rates, with a clear trend observed: higher deposition rates (10 mL/h and 7.5 mL/h) yielded greater antimicrobial effects, while lower deposition rates (5 mL/h and 2.5 mL/h) resulted in reduced antimicrobial efficacy. This trend highlights the influence of the deposition rate on the uniformity and thickness of the copper–alginate coating, which, in turn, affects the release of copper ions and the material’s overall antimicrobial performance.

Overall, these findings confirm the broad-spectrum antimicrobial activity of PET@Alg/Cu membranes and demonstrate a consistent trend of higher deposition rates correlating with greater antimicrobial efficacy across all microbial species tested. This underscores the importance of optimizing the deposition rate during fabrication to achieve the desired balance among coating uniformity, copper-ion release, and antimicrobial performance.

**Figure 5:**
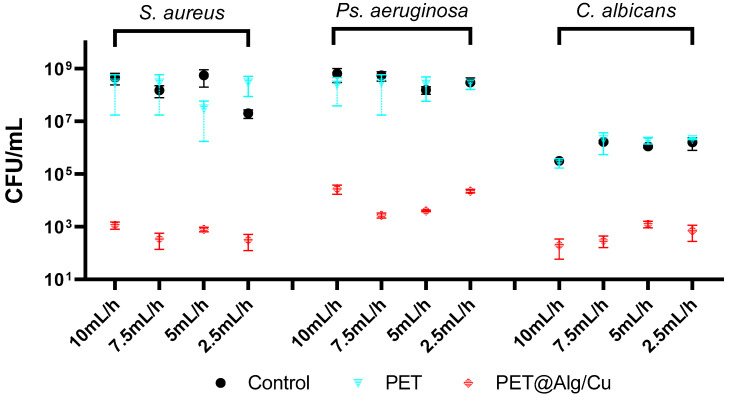
Graphical representation of CFU/mL values, illustrating the degree of adherence of *S. aureus*, *Ps. aeruginosa*, and *C. albicans* cells to the surface of the tested materials (Control, PET, and PET@Alg/Cu) following 24 h of incubation at 37 °C. PET@Alg/Cu samples.

Figure 5 illustrates log10 CFU/mL values representing microbial adherence of *S. aureus*, *Ps. aeruginosa*, and *C. albicans* to the surfaces of the tested materials (Control, PET, and PET@Alg/Cu) after 24 h of incubation at 37 °C. Control and PET-only samples showed high adherence levels for all three microbial species, with CFU/mL values consistently in the 10^7^ to 10^8^ range, indicating robust colonization on unmodified surfaces. These findings confirm the lack of inherent antimicrobial or anti-adherence properties in the PET-only materials.

In contrast, PET@Alg/Cu samples demonstrated significant reductions in microbial adherence for all three species across all deposition rates. For *S. aureus* and *P. aeruginosa*, the CFU/mL values consistently dropped to the 10^3^ range, confirming the strong anti-adherence properties conferred by the copper–alginate functionalization. This reduction was consistent across deposition rates, indicating that the fabrication process reliably imparts effective antimicrobial properties regardless of the specific electrospinning parameters. Similarly, *C. albicans* adherence was drastically reduced on PET@Alg/Cu surfaces, with CFU/mL values comparable to those observed for *S. aureus* and *Ps. aeruginosa*, demonstrating that the antifungal efficacy of the PET@Alg/Cu membranes matches their antibacterial performance. The consistent reduction in CFU/mL values across deposition rates suggests that the functionalization process is equally effective for fungal inhibition.

The antimicrobial mechanism of PET@Alg/Cu membranes likely involves a combination of copper ions (Cu^2+^) and the alginate coating, which act synergistically to inhibit microbial adherence and proliferation. Copper ions generate reactive oxygen species (ROS), disrupt microbial membranes, inhibit critical enzymes, and damage microbial DNA, resulting in effective antimicrobial activity. The alginate coating further enhances this by serving as a reservoir for copper ions, enabling their sustained release, while its hydrophilic properties discourage microbial colonization. Together, these features ensure the PET@Alg/Cu membranes’ broad-spectrum antimicrobial efficacy, making them effective at preventing microbial adherence and biofilm formation across diverse microbial species.

**Figure 6:**
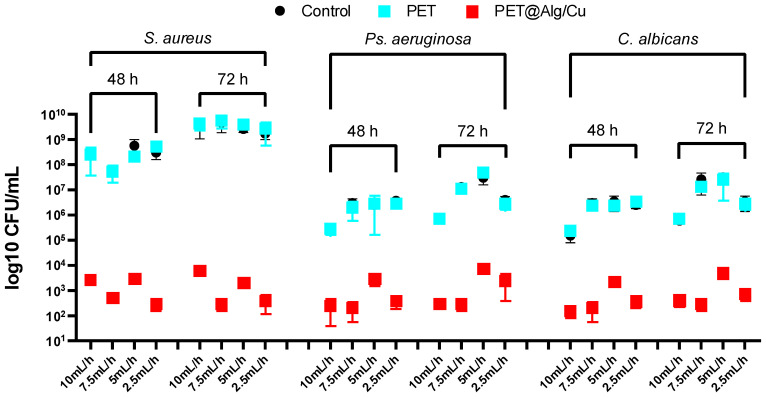
Log10 CFU/mL values showing biofilm formation by *S. aureus*, *Ps. aeruginosa*, and *C. albicans* on Control, PET, and PET@Alg/Cu materials after 48 and 72 h at 37 °C.

Figure 6 presents log10 CFU/mL values, showing biofilm formation by *S. aureus*, *Ps. aeruginosa*, and *C. albicans* on Control, PET, and PET@Alg/Cu materials after 48 and 72 h at 37 °C. For Control and PET-only samples, biofilm formation remained consistently high for all three microbial species, with CFU/mL values ranging from 10^7^ to 10^9^. These findings are consistent with the 24 h results, where the same materials showed high microbial adherence levels (10^7^–10^8^), demonstrating that the unmodified surfaces of PET samples allow the transition from microbial adhesion to robust biofilm development. This highlights their lack of inherent anti-biofilm properties, as the biofilms continue to mature and persist over time.

In contrast, PET@Alg/Cu samples exhibited significant reductions in biofilm formation across all microbial species and time points. At 24 h, PET@Alg/Cu materials reduced microbial adherence to 10^3^ CFU/mL, effectively halting the initial stages of biofilm formation. These results were mirrored at 48 and 72 h, at which times the biofilm CFU/mL values remained in the 10^3^–10^4^ range for *S. aureus*, *Ps. aeruginosa*, and *C. albicans*. This consistent 5–6 log reduction across both early and later stages of biofilm formation underscores the effectiveness of the copper–alginate functionalization in preventing the development and maturation of biofilms, regardless of deposition rate.

The strong antifungal activity of PET@Alg/Cu materials against *C. albicans* further supports these findings. The reduction of CFU/mL values to 10^3^–10^4^ across all time points matches the reductions observed for bacterial biofilms, indicating broad-spectrum anti-biofilm efficacy. By correlating these results with the 24 h data, it is evident that PET@Alg/Cu disrupts multiple stages of the biofilm lifecycle, from initial microbial adhesion to microcolony formation and biofilm maturation.

**Figure 7:**
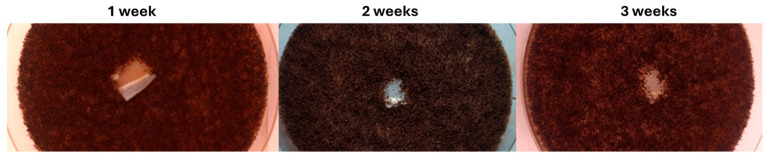
Development of *A. niger* cultures in the presence of fibrillar membranes composed of PET and PET@Alg/Cu (2.5 mL/h) after 1, 2, and 3 weeks of incubation.

The antifungal evaluation demonstrates that PET@Alg/Cu membranes effectively inhibit the growth of *A. niger* on their surface for at least three weeks (Figure 7). This inhibitory effect is particularly pronounced, as evidenced by the visibly reduced fungal growth in the areas surrounding the PET@Alg/Cu membranes. These findings highlight the robust and sustained antifungal properties of PET@Alg/Cu membranes, further supporting their potential for long-term applications in environments prone to filamentous fungal contamination, such as industrial facilities, healthcare settings, or food storage environments.

### 3.3. Cytotoxicity Evaluation

**Figure 8:**
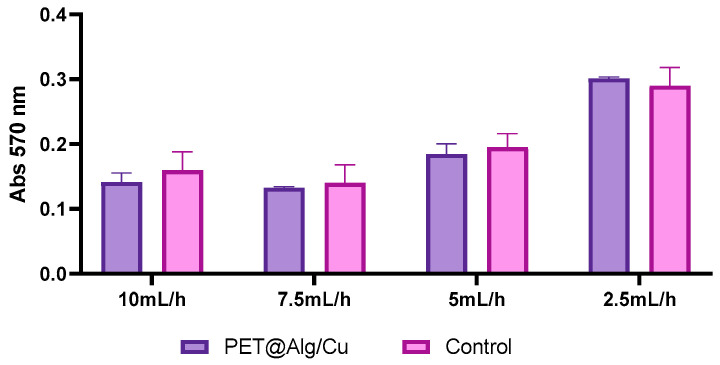
Graphical representation of MTT assay results showing absorbance values at 570 nm, indicating the optical density of formazan produced by mitochondrial oxidoreductase activity and reflecting the metabolic activity and proliferation of diploid cells in the presence of PET and PET@Alg/Cu materials at different deposition rates.

The MTT assay results in Figure 8 indicate that the PET@Alg/Cu materials support cellular metabolic activity, as shown by absorbance values at 570 nm reflecting mitochondrial oxidoreductase activity. Across the tested deposition rates, no significant reduction in cell viability is observable compared to the control. The samples exhibit absorbance values closely matching the control, indicating excellent biocompatibility and minimal interference with cellular metabolic processes.

These findings demonstrate that the PET@Alg/Cu materials, regardless of deposition rate, maintain biocompatibility and do not adversely affect diploid cell viability. Variations in cell activity between deposition rates may reflect subtle differences in material properties, such as surface structure or composition. This indicates the potential of PET@Alg/Cu materials as promising candidates for applications requiring biocompatible surfaces, such as in tissue engineering or biomedical implants.

### 3.4. GSH Test Results

**Figure 9:**
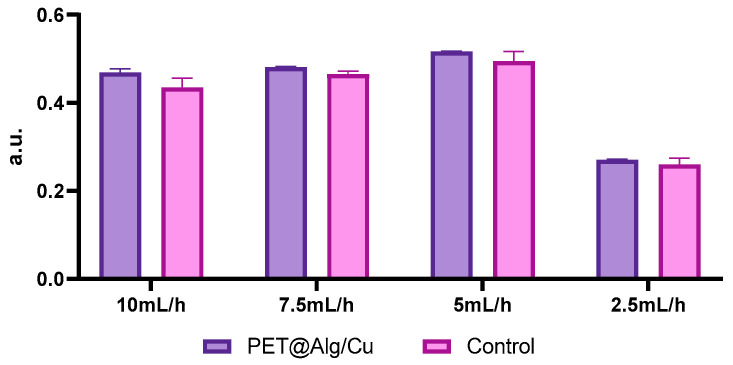
Graphical representation of GSH assay results, expressed in arbitrary units (a.u.), indicating the activity of glutathione S-transferase (GST) of diploid cells cultured in the presence of PET@Alg/Cu materials at different deposition rates compared to the control.

The GSH assay results in Figure 9 reveal the activity of glutathione S-transferase (GST) as a measure of oxidative stress in cells exposed to PET@Alg/Cu materials at different deposition rates. The luminescence values, expressed in arbitrary units (a.u.), indicate that the materials consistently maintain oxidative balance comparable to the control under all tested conditions. Across all deposition rates (10 mL/h, 7.5 mL/h, 5 mL/h, and 2.5 mL/h), the GST activity for PET@Alg/Cu is nearly identical to that of the control, suggesting minimal induction of oxidative stress and excellent biocompatibility.

These findings demonstrate that deposition rate does not significantly impact oxidative stress response, highlighting the robustness and reliability of PET@Alg/Cu materials. This consistency further confirms their suitability for applications requiring biocompatibility and controlled cellular interactions.

### 3.5. In Vivo Biocompatibility

**Figure 10:**
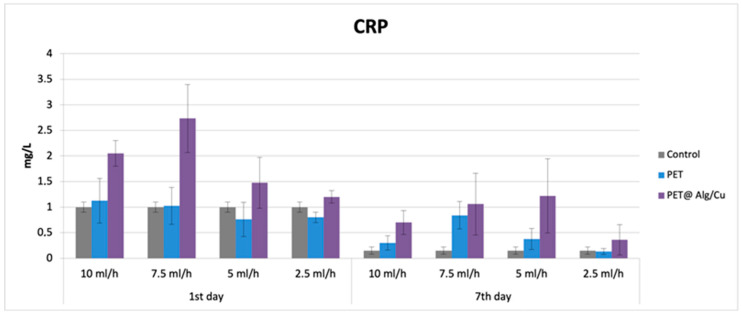
The effects of subcutaneous PET@Alg/Cu implantation in mice on the C-reactive protein (CRP) levels at 24 h and 7 days post-surgery.

Activity of C-reactive protein (CRP) inflammatory marker. Figure 10 shows the effects of the subcutaneous implantation of PET@Alg/Cu on the serum level of the inflammatory marker CRP. At 24 h after implantation, serum CRP level was raised for all experimental groups, followed by a 14-day decrease. For both time intervals, the CRP level was significantly increased for the PET@Alg/Cu group compared to the Control and PET groups, respectively.

### 3.6. Histology

The post-implantation clinical evaluation revealed the appearance of a slight reddening of the skin for the PET@Alg/Cu group. The other experimental groups did not show similar clinical signs.

**Figure 11:**
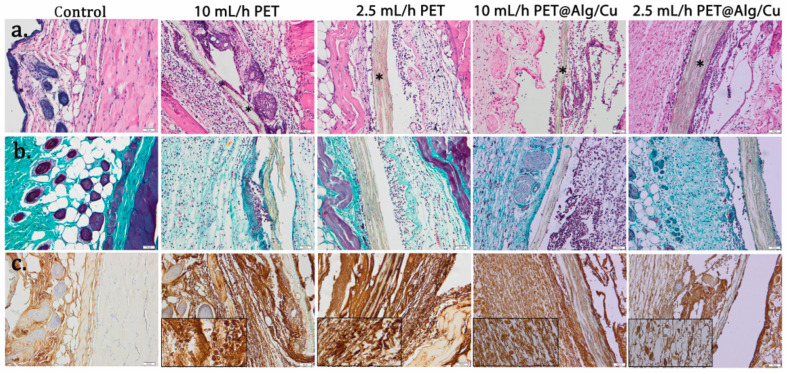
Histopathological analysis of PET@Alg/Cu at 24 h post-implantation. (**a**) H&E stain; (**b**) TNF-α immunohistochemistry; (**c**) Masson–Goldner trichrome stain; (*) material; bars 50 μm and 20 μm (details).

Histological analysis of the PET group revealed edema around the material at 24 h, which was maintained at 7 days after surgery (Figure 11a). This reaction increased with the rate of fiber deposition. At 24 h after implantation, the presence of an inflammatory infiltrate in the cutis and subcutis, consisting mainly of polymorphonuclear neutrophils (PMN) neutrophils, was observed (Table 1). The inflammatory infiltrate was present in the histological sections of the PET@Alg/Cu group, while a significant number of eosinophils in the connective tissue and extravasated red blood cells from the capillaries were observed (Figure 11a; Table 1).

**Figure 12:**
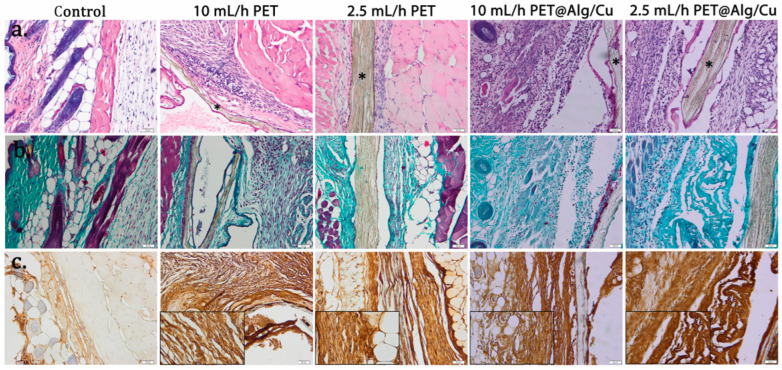
Histopathological analysis of PET@Alg/Cu at 7 days post-implantation. (**a**) H&E stain; (**b**) TNF-α immunohistochemistry; (**c**) Masson–Goldner trichrome stain Material (*); bars 50 μm and 20 μm (details).

After 7 days, collagen proliferation, which promotes the formation of a fibrous capsule around the materials, was noticed (Figure 12b). Alginate-coated PET materials are surrounded by connective tissue consisting of fibroblasts, many macrophages, eosinophils, and proliferating capillaries.

Immunohistochemistry was performed to analyze inflammatory response towards the implanted materials. As shown in Figure 11c and Figure 12c, immunopositivity for TNF-α increased in connective tissue surrounding PET materials in a time-dependent manner. The immunoreaction was stronger for PET coated with alginate than for PET or the control.

**Figure 13:**
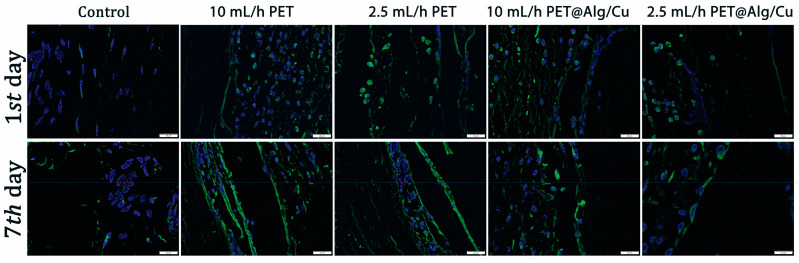
F4/80 protein expression as revealed by confocal microscopy at 24 h and 7 days post-implantation. F4/80 is labeled in green, and the nuclei are counterstained with DAPI; magnification ×63.

The F4/80 marker was positive in all samples with subcutaneously implanted material, highlighting the presence of macrophages in the peri-implant connective tissue, especially 7 days after surgery (Figure 13). At 7 days post-implantation, the activated macrophages bordered the material and were highly positive for F4/80.

## 4. Discussion

This study aimed to obtain and characterize novel composite materials based on chemically recycled and electrospun PET with improved biological properties to prevent microbial contamination. Morphostructural investigations revealed that the obtained materials were randomly oriented, with many curvatures along the fiber axis and diameters ranging from 50 to 200 nm, depending on the mass ratios used. SEM analyses also allowed visualization of the alginate layer deposited on PET@Alg/Cu samples, confirming the successful functionalization of recycled PET membranes. The presence of copper alginate was also reflected in FT-IR spectra through characteristic absorption bands. Moreover, FT-IR spectra proved that the chosen fabrication method maintained PET characteristics, as the chemical structure of the polymer was not altered after electrospinning. Further, given the intended application of the obtained materials, in-depth biological characterization was performed to assess the antimicrobial potential and biocompatibility of the recycled PET-based membranes.

Nosocomial infections (also known as healthcare-associated or hospital-acquired infections) are recurrent problems mainly identified in intensive care facilities, as well as surgical and medical wards [54,55,56,57]. Hence, our research focused on finding modern solutions to combat these microorganisms and creating fibrous membranes with anti-infective effects against relevant hospital-acquired pathogens. Specifically, the fabricated materials were tested against *S. aureus*, *P. aeruginosa*, *C. albicans*, and *A. niger*, leading to encouraging results for their future application.

Microbial cultures develop differently and exhibit distinct phenotypic and molecular adaptations in the planktonic state compared to those growing in the adherent state. Comparative studies on planktonic and adherent cultures demonstrate that adherent microorganisms forming multicellular communities, called biofilms, exhibit varying degrees of resistance, virulence, and typical metabolic particularities. The ability to adhere to a biotic or abiotic surface represents the first stage in initiating an infectious process or in colonizing various surfaces [58,59,60,61,62,63]. Thus, it is essential to focus on developing materials that are able to inhibit microbial colonization and prevent biofilm formation on their surfaces to mitigate infection risks. Therefore, new methods are being studied to prevent contamination with microorganisms and strategies to limit microbial adherence and inhibit biofilm development.

In this context, this study proposes a threefold approach by functionalizing PET fibers with a layer of a biocompatible natural polymer (i.e., alginate) enhanced with metal with intrinsic antimicrobial properties (i.e., copper). This functionalization imparts the material with enhanced antimicrobial activity and biocompatibility, which is crucial for wound dressing applications. To our knowledge, the specific combination of these three components (rPET, alginate, and copper) has not been reported in nanofibrous wound dressings.

The biological test results obtained in this study suggest that fibrous membranes made from recycled PET and non-toxic antimicrobial agents produced through electrospinning hold significant promise as a novel strategy for developing advanced wound dressing materials. The fabricated PET@Alg/Cu nanofibrous membranes demonstrated remarkable efficiency in inhibiting potentially pathogenic microorganisms’ colonization and biofilm formation while maintaining excellent biocompatibility, making them suitable for healthcare applications. Moreover, this study systematically investigated the effect of different electrospinning deposition rates on fiber morphology, antimicrobial performance, and biocompatibility, uncovering new insights into advancing functional nanofibers for medical applications. Future research may also consider the advantages of digital tools for further optimization stages, with predictive computational models and machine learning algorithms holding great promise to improve deposition parameters for given target material properties [64,65,66,67,68,69].

Recent literature findings have also revealed the usefulness of fiber functionalization with other biocompatible polymers. The reported possibilities include PET nanofibers modified with chitosan [70,71,72], polycaprolactone [73], cellulose diacetate [74], and silk sericin [75]. Several studies on PET fibers have also focused on conferring antimicrobial activity upon this synthetic polymer by incorporating various nanomaterials. Specifically, PET nanofiber functionalization agents include silver nanoparticles [75,76,77,78], usnic acid-functionalized magnetic nanoparticles [30], nickel nanoparticles [79], magnesium oxide nanoparticles [80], zinc oxide nanoparticles [81], and Cu_2_O@ZrP nanosheets [82]. With so many alternative options, future research should aim to compare the cost and clinical performance of PET@Alg/Cu membranes against other nanomaterial-based functionalization approaches. Such analyses would provide a clearer understanding of the economic feasibility and potential advantages of PET@Alg/Cu membranes in clinical and large-scale applications.

This study aligns with the latest PET-based composite nanofiber development trends, introducing a novel composition with significant potential for future applications in advanced wound dressing materials. In addition, the multifunctionality of PET@Alg/Cu membranes suggests potential for broader use in biomedical fields, such as drug delivery and tissue engineering scaffolds. For instance, alginate’s biocompatibility, coupled with copper’s antimicrobial properties, may enable controlled drug release over time, reducing infection risks while promoting targeted therapies [83,84]. Moreover, the fibrous architecture of the membranes could provide structural support for cell adhesion and growth, making them suitable for tissue scaffolding [85,86,87]. Future work should explore these directions to fully realize the versatility of the newly developed materials in advanced healthcare solutions.

Thus, our research bridges sustainability and nanotechnology by utilizing post-consumer PET waste while addressing significant clinical needs, such as preventing biofilm-associated infections. While the current study focuses on developing and characterizing recycled PET-based nanofibers, a comprehensive Life Cycle Assessment (LCA) is necessary to evaluate their actual sustainability benefits. Preliminary estimates from the literature suggest significant environmental gains through the reuse of PET [88,89]. However, these remain to be rigorously validated in nanofiber production, where parameters such as greenhouse gas emissions, energy consumption, and the economic viability of scaling up recycled PET applications must be compared with the parameters of virgin PET. Such assessments are planned in future work.

## 5. Conclusions

This study aimed to synthesize and characterize, from a physicochemical and biological perspective, fibrous membranes based on recycled PET and alginate functionalized with CuCl_2_, obtained through the electrospinning technique, with a focus on their potential as advanced wound dressing materials. The obtained materials demonstrated desirable physicochemical properties, such as organized morphology, suitable dimensions, and structural integrity, which recommend them for medical applications. Biological evaluations showed that rPET-based membranes effectively inhibit microbial adherence and colonization while exhibiting low cytotoxicity toward diploid eukaryotic cells in culture. Furthermore, the antimicrobial and biological effects were found to depend on the fiber deposition rate during the electrospinning process. Materials coated with alginate and CuCl_2_ showed significant antimicrobial and anti-biofilm activity, effectively limiting the growth of planktonic microorganisms and the development of monospecific biofilms, particularly for *S. aureus*, *P. aeruginosa*, and *C. albicans*. Cytotoxicity tests (MTT and GSH) indicated that while the materials could influence cell proliferation and metabolic activity, they did not induce cell death under most tested conditions, except for certain deposition parameters. In conclusion, optimizing electrospinning protocols, particularly the deposition rate, is essential for tailoring these materials for wound dressing applications. The developed fibrous membranes have strong potential for use as wound dressings, offering antimicrobial properties and biocompatibility to reduce infection risks and support healing in clinical environments.

## Figures and Tables

**Table 1:** Tissue reactions by histometric scoring used to grade inflammation and neovascularization in the tissue surrounding subcutaneous implants.

Material	Implantation Period (Days)	Edema	PMN	M	E	RBC	F	NV
Control	1	-	+	-	-	-	-	-
	7	-	-	+	-	-	-	-
PET 10 mL/h	1	++++	+++	++	+	-	+	-
	7	+++	++	+++	+	-	++++	-
PET 7.5 mL/h	1	+++	+++	+	+	-	+	-
	7	++	+	+++	+	-	+++	-
PET 5 mL/h	1	++	+++	+	+	-	+	-
	7	++	+	+++	+	-	++	+
PET 2.5 mL/h	1	++	+++	+	+	-	+	-
	7	+	+	+++	+	-	++	+
PET@Alg/Cu 10 mL/h	1	++	++++	++	+++	++	+	-
	7	+	+	++++	+++	+	++++	+
PET@Alg/Cu 7.5 mL/h	1	++	+++	+	+++	++	+	-
	7	+	+	++++	+++	+	++++	+
PET@Alg/Cu 5 mL/h	1	+	+++	+	++	++	+	-
	7	+	+	+++	++	+	+++	+
PET@Alg/Cu 2.5 mL/h	1	+	+++	+	++	+	+	-
	7	-	+	+++	++	+	+++	+

PMN: polymorphonuclear neutrophils; M: macrophages; E: eosinophils; RBC: extravasated red blood cells; F: fibroblasts, NV: neovascularization. Tissue reactions are rated from - (not present) to ++++ (extensive).

## Data Availability

The original contributions presented in this study are included in the article. Further inquiries can be directed to the corresponding author.
